# miR-127 accelerates myocardial failure by promoting TGF-β1/Smad3 signaling

**DOI:** 10.3892/mmr.2019.10442

**Published:** 2019-06-28

**Authors:** Hainian Xu, Fengmei Li

Mol Med Rep 18: 4839-4846, 2018; DOI: 10.3892/mmr.2018.9514

After the publication of the above article, the authors have found that they are not able to reproduce certain of the results presented in their research. The authors have expressed their concern about the choice of cell line selected for their original experiments, which may account for why their results are no longer reproducible. As a consequence, the authors have requested that this article be retracted from the publication.

Both authors on the paper agree to the retraction, and regret the inconvenience that this retraction will cause to the Editor and to the readership of the Journal.

